# The emergence of high room temperature in-plane and out-of-plane magnetostriction in polycrystalline CoFe_2_O_4_ film

**DOI:** 10.1038/s41598-021-02421-w

**Published:** 2021-11-24

**Authors:** Suman Guchhait, H. Aireddy, A. K. Das

**Affiliations:** 1 Department of Physics, Indian Institute of Technology Kharagpur, Kharagpur, 721302 India; 2 Department of Electronics and Communication Engineering, Alliance College of Engineering and Design, Bengaluru, 560076 India

**Keywords:** Materials science, Nanoscience and technology, Physics

## Abstract

The polycrystalline CoFe_2_O_4_ (CFO) film on cantilever substrate of silicon was grown using pulsed laser deposition (PLD) method and investigated its in-plane and out-of-plane magnetostrictive strain at room temperature (300 K) using the indigenous optical Cantilever Beam Magnetometer (CBM). The film shows a high compressive magnetostrictive strain of ‒ 387 ppm and ‒ 708 ppm for in-plane and out-of-plane configurations, respectively. Considerably, the magnetostrictive strain loops (λ‒H) possess a certain degree of hysteresis with a symmetric butterfly shape. The origin of large compressive magnetostriction of CFO film is attributed to the non-180° domain wall motion followed by 90° domain rotation. The large values of saturation magnetostrictive strain make CFO film a suitable candidate in sensor design for different purposes.

## Introduction

Magnetostriction is the deformation (expansion or compression) of magnetic materials in the presence of the magnetic field^1^. The so-called deformation arises because of the intimate coupling between the magnetic moment and the crystal lattice^2^. The nature (tensile or compressive) of deformation is governed by the sign (+/‒) of magnetostrictive strain (λ). Theoretically, the strain varies quadratically with the magnetization of the concerned material^3^, i.e., λ ~ ${M}^{2}$. Nowadays, magnetostrictive materials are widely used for making the different types of sensors^4,5^, actuators^6,7^, motors^8^, transducers^9^, etc. Not only the magnitude but also the sign (+/‒) of the strain are important parameters, which define the potentiality of a particular material for device fabrication. The high value of the magnetostrictive strain is always desirable for the devices, but its sign makes it suitable for a specific application. The materials having positive strain are suitable for actuators, while negative magnetostrictive materials are preferable for sensor design^10^. The research on magnetostrictive films has got much attention from the mid-1970s^11^, and many groups have reported large magnetostriction of different multilayer and monolayer systems till the date^12–18^. The thin films of Fe-based rare-earth alloys, including Tb–Dy–Fe (magnetostrictive strain over 1000 ppm), exhibit giant magnetostriction and thereby easily meet the criteria for the fabrication of sensors and microactuators in micro-electro-mechanical systems (MEMS)^11^. But the several factors like high magnetocrystalline anisotropy, lack of rare-earth materials, brittleness, cost, etc. limit their uses in application purposes^19^. This inspires us to enquire into well-performing yet sustainable alternative magnetostrictive materials. The use of oxide-based magnetostrictive materials, particularly cobalt ferrite (CFO) has received much attention due to its large magnetostriction, high strain sensitivity, lower saturation field, easy to fabricate, and economically cheap^20,21^. CFO crystallizes in inverse type cubic spinel structure with space group Fd$\overline{3 }$m (no. 227)^22^. Bozorth et al. reported magnetostriction, ${\lambda }_{100}$ ~ ‒ 515 ppm for single-crystal CFO^23^. Chen et al. obtained saturation magnetostriction, ${\lambda }_{s}$ ~ ‒ 225 ppm for a polycrystalline sample^24^. Muhammad et al. showed magnetostriction, ~ ‒ 400 ppm for a sintered pellet under high pressure of 150 MPa^25^. In this article, we report the room temperature magnetostriction in the in-plane and out-of-plane configurations of polycrystalline CFO film characterizing with an indigenously built optical cantilever beam magnetometer (CBM) set-up. We have achieved high magnetostriction in both configurations and there is a significant enhancement of magnetostriction in the out-of-plane geometry relative to that of the in-plane geometry. The detailed procedure of the measurement using the CBM set-up has been described elsewhere^26^.

## Results and discussion

**Figure 1:**
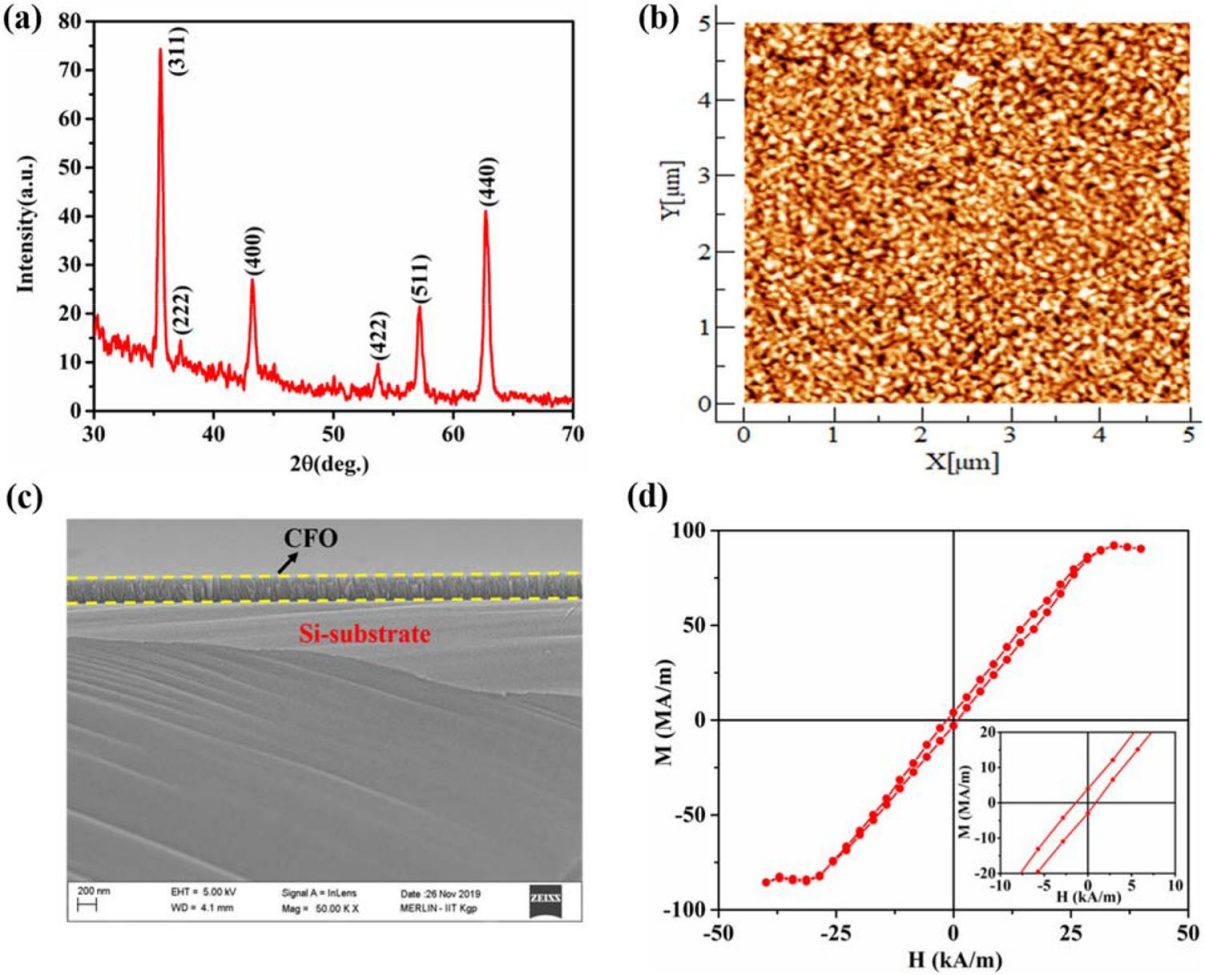
(**a**) GIXRD pattern of CFO film. (**b**) AFM image of CFO film over (5 μm × 5 μm) scan area. (**c**) Cross-sectional FESEM view of CFO/Si heterojunction. (**d**) The M–H curve of CFO film at 300 K.The magnified views of the hysteresis loop is shown at the inset.

**Figure 2:**
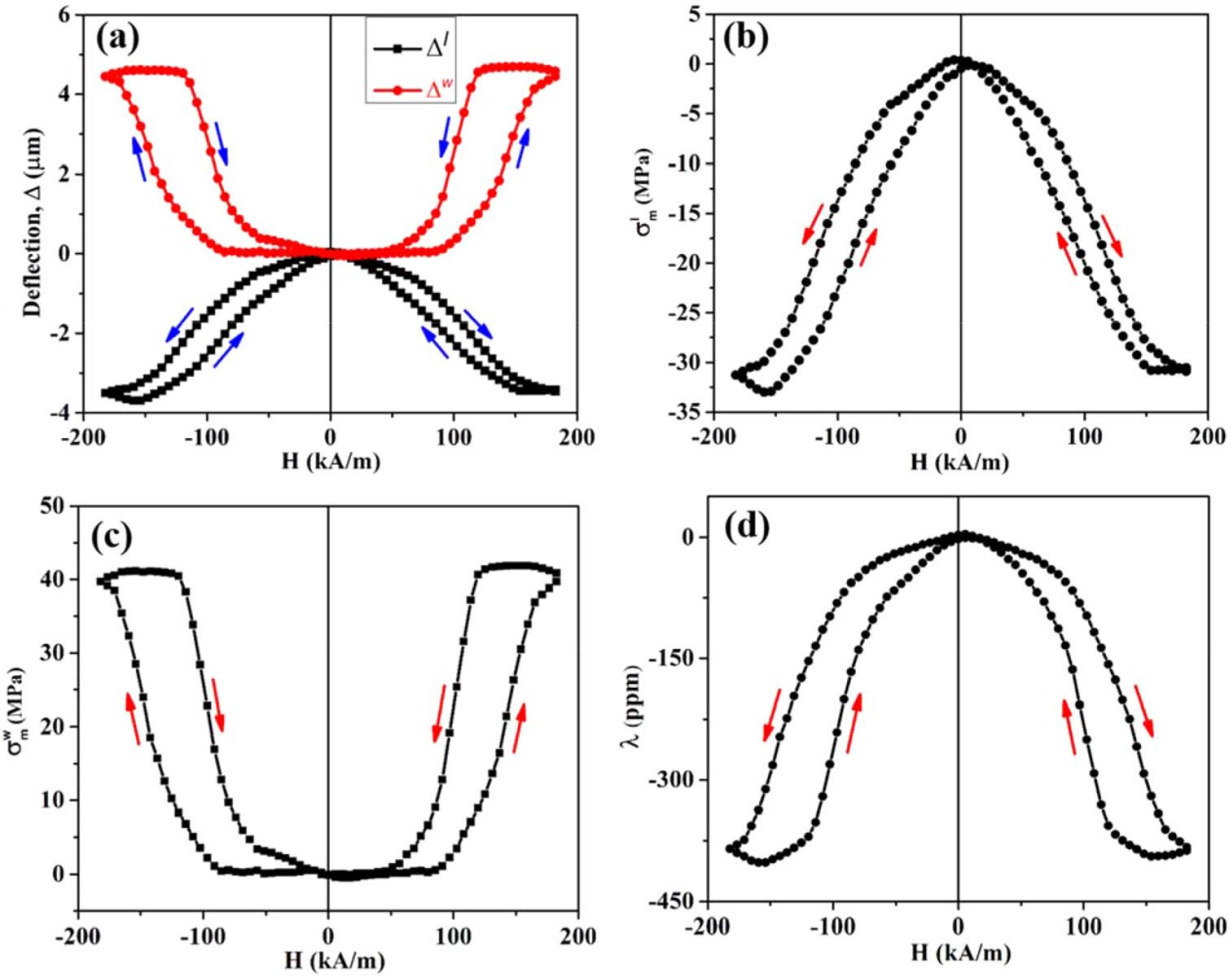
(**a**) The CFO/Si deflections of the free end of the cantilever substrate with the applied field along the length and along the width of the film. (**b**) In-plane magnetostrictive stress developed in the sample when the magnetic field is applied along the length of the film. (**c**) Magnetostrictive stress when the field is along the width of the film. (**d**) The variation of in-plane magnetostriction with the magnetic field.

**Figure 3:**
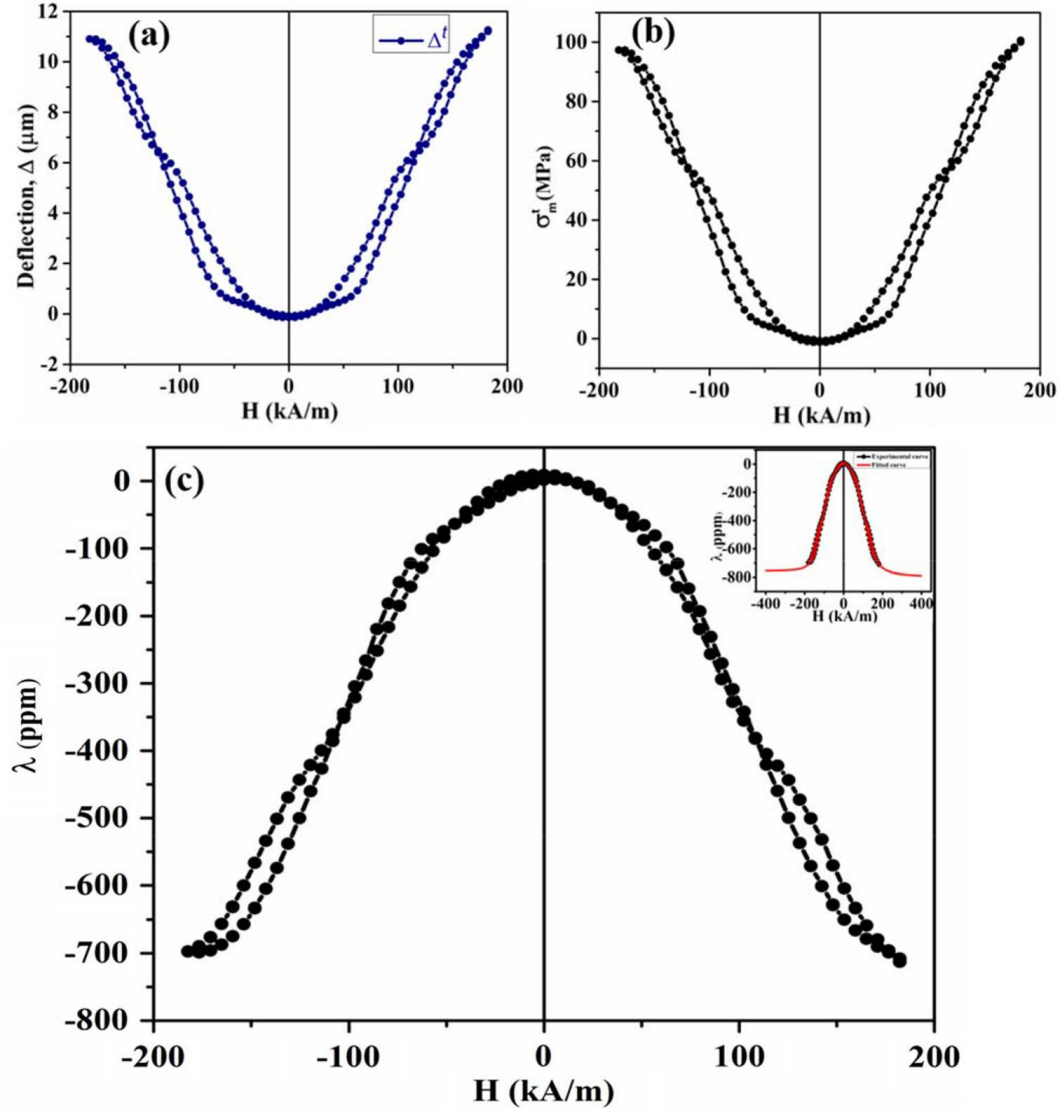
(**a**) The plot of cantilever deflection versus the applied magnetic field of CFO/Si. (**b**) The variation of out-of-plane magnetostrictive stress with the magnetic field. (**c**) The out-of-plane (λ‒H) plot, inset shows the fitted and extrapolated curve along with the experimental curve.

The Grazing Incidence X-ray Diffraction (GIXRD) profile of the CFO film over the specified angular range has been shown in Fig. 1a. The profile indicates that the deposited film is polycrystalline. The observed peaks are indexed with the help of a standard JCPDS file (JCPDS card. no. 22-1086), and it reveals the formation of a single-phase with a cubic spinel structure. The 2D topography of the film over the selected area through the Atomic Force Microscope (AFM) study has been depicted in Fig. 1b. The root mean square (r.m.s) surface roughness value is found to be 0.86 nm over the scanned area. The low roughness value suggests that the surface of the film is quite smooth. The cross-sectional field emission scanning electron microscopy (FESEM) image of CFO/Si heterojunction taken at a magnification of 50,000 (50 k) has been shown in Fig. 1c. The typical cross-sectional view ensures the growth of CFO film over the Si substrate. The average thickness of the CFO film is calculated to be ~ 260 nm. The typical M–H curve at room temperature has been shown in Fig. 1d. The hysteresis nature of the M–H loop can be visualized from the zoomed view shown at the inset of Fig. 1d. The saturation magnetization (${M}_{s}$) is ~ 90.27 MA/m, whereas the remanent magnetization (${M}_{r}$) and coercive field (${H}_{c}$) are obtained to be ~ 3.49 MA/m and ~ 1.13 kA/m, respectively from the hysteresis curve. In in-plane geometry, the deflections of the free end of the cantilever substrate of sample/substrate composite as a function of the applied magnetic field have been represented in Fig. 2a. For applied field along the length of the film, the maximum deflection (${\Delta }^{l}$) is ~ ‒ 3.45 μm and for the field along the width of the film, the maximum deflection (${\Delta }^{w}$) is ~  + 4.69 μm. The variations of in-plane magnetostrictive stresses with the applied bipolar field are shown in Fig. 2b,c. Here, the “red arrows” indicate the sweep directions of the applied field. The curves are butterfly-shaped and having a certain degree of hysteresis. It has been observed that ${\sigma }_{m}^{l}$ is compressive and ${\sigma }_{m}^{w}$ is tensile in nature. The maximum compressive stress has been found to be ~ ‒ 31 MPa, whereas the maximum tensile stress developed in the sample, is ~  + 41 MPa. The dependence of in-plane magnetostriction with the applied magnetic field is shown in Fig. 2d. It shows a butterfly type parabolic hysteresis loop, which is compressive in nature. We have got the maximum value of magnetostriction to be ~ ‒ 387 ppm from the (λ‒H) plot. Figure 3a represents the variation of deflection of CFO/Si composite with the applied bipolar magnetic field in the out-of-plane geometry. The maximum deflection is found to be ~  + 11.20 μm. The magnetic field dependence of out-of-plane magnetostrictive stress, as well as magnetostriction, is shown in Fig. 3b,c, respectively. Note that the reference direction of magnetization was taken in-plane along the length of the cantilever, with respect to which the out-of-plane magnetostriction along the thickness has been calculated. It has been observed that in both cases (Fig. 3b,c), we are getting butterfly-type hysteresis loops, but they are different in nature. Here, the former one is tensile, whereas the latter one is compressive. The maximum stress is determined to be ~  + 100 MPa, and the maximum value of the magnetostriction is ~ ‒ 708 ppm. In the out-of-plane configuration, due to the shape anisotropy of the film, higher value of the field is required to get the saturation behavior. Unfortunately, the maximum field is limited to ~  ± 199 kA/m due to large pole gap for placing the sample assembly. However, we have fitted the magnetostriction data and extended to higher magnetic field (~ ± 398 kA/m). One can see a clear saturation of magnetostriction at magnetic field of ~  ± 358 kA/m. The value of the saturation magnetostriction is estimated to be ~ ‒ 754 ppm. The variation of corresponding magnetostriction vs magnetic field is shown as inset of Fig. 3c. Here, the “black” curve represents the experimental curve and the “red” curve is the fitted one.

In this work, we have obtained high values of magnetostrictions for the in-plane as well as the out-of-plane configuration of the polycrystalline CFO film at room temperature. The (λ‒H) curves are compressive in nature in both configurations. The magnetostrictive hysteresis loops have the conventional butterfly shape and exhibit approximately the reflection symmetry about H = 0 axis. Also, the parabolic shape of the hysteresis loops signifies that the magnetostriction (λ) varies quadratically with magnetization (M) of the CFO film, i.e., λ ~ ${M}^{2}$. The origin of the magnetostriction of CFO film lies under the fact of deformation of non-180° domains followed by domain magnetization rotation^27^. The 180° domains are not stress-sensitive. Therefore, they do not take part in the magnetostriction phenomenon. But the non-180° domains are stress-sensitive and are responsible for the origin of magnetostriction^28^. The deformation of non-180° domains can happen in two ways; one is the reversible domain wall (DW) displacement, and the other one is the irreversible domain wall displacement^24,29^. The nature of the DW displacement depends on the strength of DW pinning, as well as on the magnitude of the applied field^24^. Suppose, the domain walls are strongly pinned at certain points and are free to move in between them. Now, at a low field, the domain walls act as an elastic membrane. If we withdraw the field, the domain walls return to their initial pinning sites and thus give rise to reversible DW motion, which is observed at the very initial part of (λ‒H) curves. But for a relatively high magnetic field, the domain walls move from one pinning site to another, and upon withdrawal of the field, they don't return to the initial pinning sites. This causes an irreversible change in domain magnetization and thereby leads to the hysteretic behavior of (λ‒H) curves^24,28^. At much higher field strength, the domain magnetization rotation takes place, which causes saturation in both loops^27^. To understand the compressive nature of the magnetostrictive strain curves in both configurations, we have to concentrate on the nature of magnetostrictive stresses developed on the film due to the change in directions of the magnetic field. When we have applied the field along different directions, magnetostrictive stress has been developed on the film due to the converse piezomagnetic effect. It has been mentioned earlier that when the magnetic field is applied along the length of the film, the developed magnetostrictive stress is compressive in nature. But for the other two mutually perpendicular field directions (along width and thickness), the magnetostrictive stresses are tensile in nature. When we are applying the magnetic field along the length, the magnetic domains oriented at angles more than 90° with respect to the field direction will try to orient themselves along the field through 90° domain rotation. As a result, the shape of the sample gets contracted, and this gives rise to the compressive stress in the film^30^. But for the rest of the two mutually perpendicular field directions (along width and thickness), the domain vectors are rotated towards the axes, and this leads to the elongation in domain shape. This magnetic domain elongation is responsible for the evolution of tensile nature in magnetostrictive stress^31^. Since we have calculated the magnetostriction by subtracting ${\sigma }_{m}^{w} \mathrm{and} {\sigma }_{m}^{t}$ from ${\sigma }_{m}^{l}$, the tensile nature of ${\sigma }_{m}^{w}$ & ${\sigma }_{m}^{t}$ relative to ${\sigma }_{m}^{l}$ (compressive) is responsible for the evolution of compressiveness in the magnetostrictive strain loops in both geometries. The CFO film could be used in different aspects based on the area of the (λ‒H) loops. In the in-plane geometry, the area of the hysteresis loop is considerably bigger and hence would be useful in fabricating memory devices such as magnetostrictive delay lines, ferroacoustic memories, etc.^32^. But in the out-of-plane geometry, we have achieved a smaller area of the loop which signifies a considerable reduction in energy loss. Therefore, the film could be employed in making transformer cores as inductive components.

**Figure 4:**
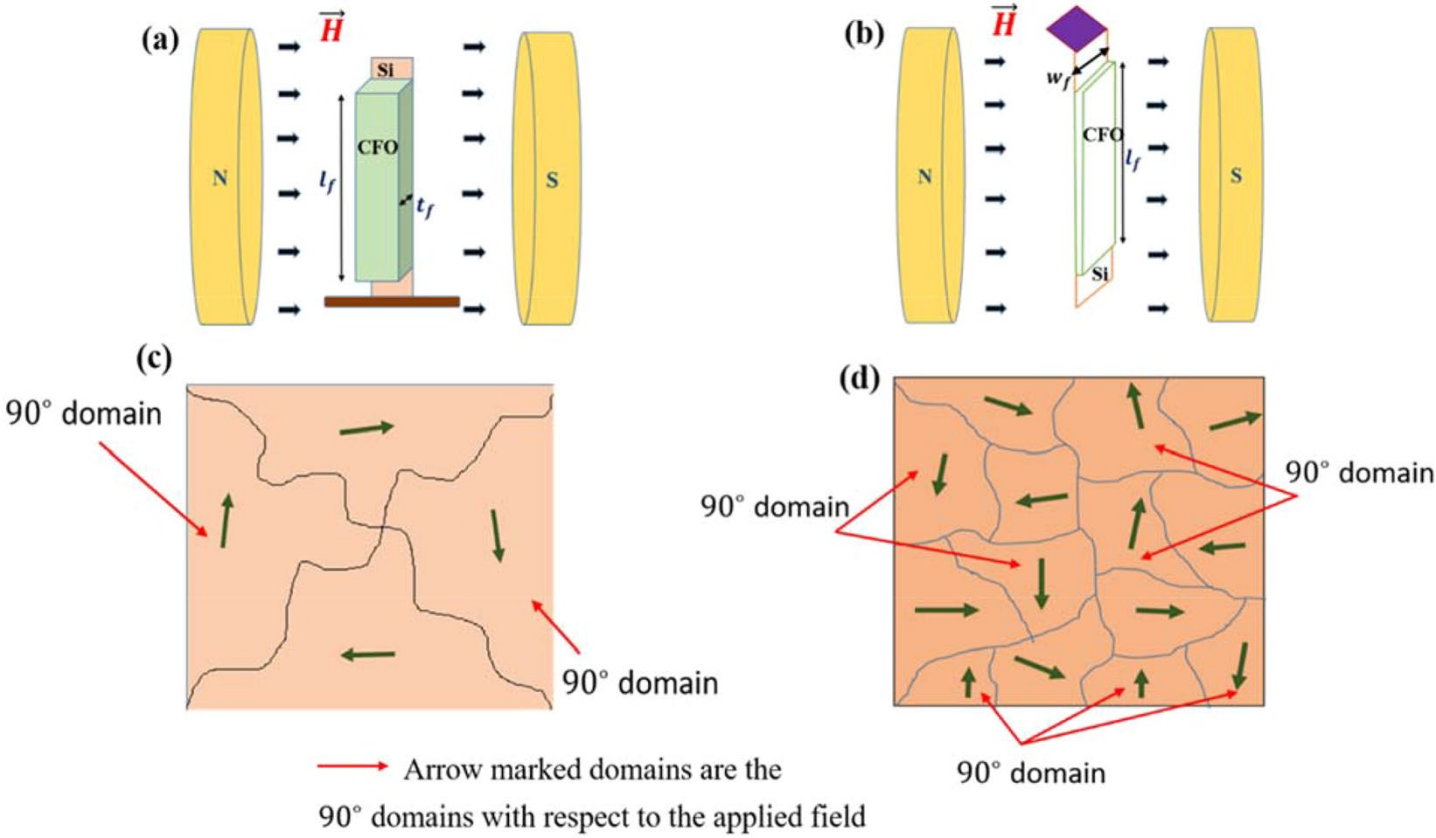
(**a**) The schematic view of CFO/Si composite when the field is applied along the width. (**b**) The schematic representation for the field is applied along the thickness. (**c**) The exposed film surface in in-plane geometry consisting of less number of 90° domains. (**d**) The exposed surface area in out-of-plane geometry having a large no. of 90° domains.

**Figure 5:**
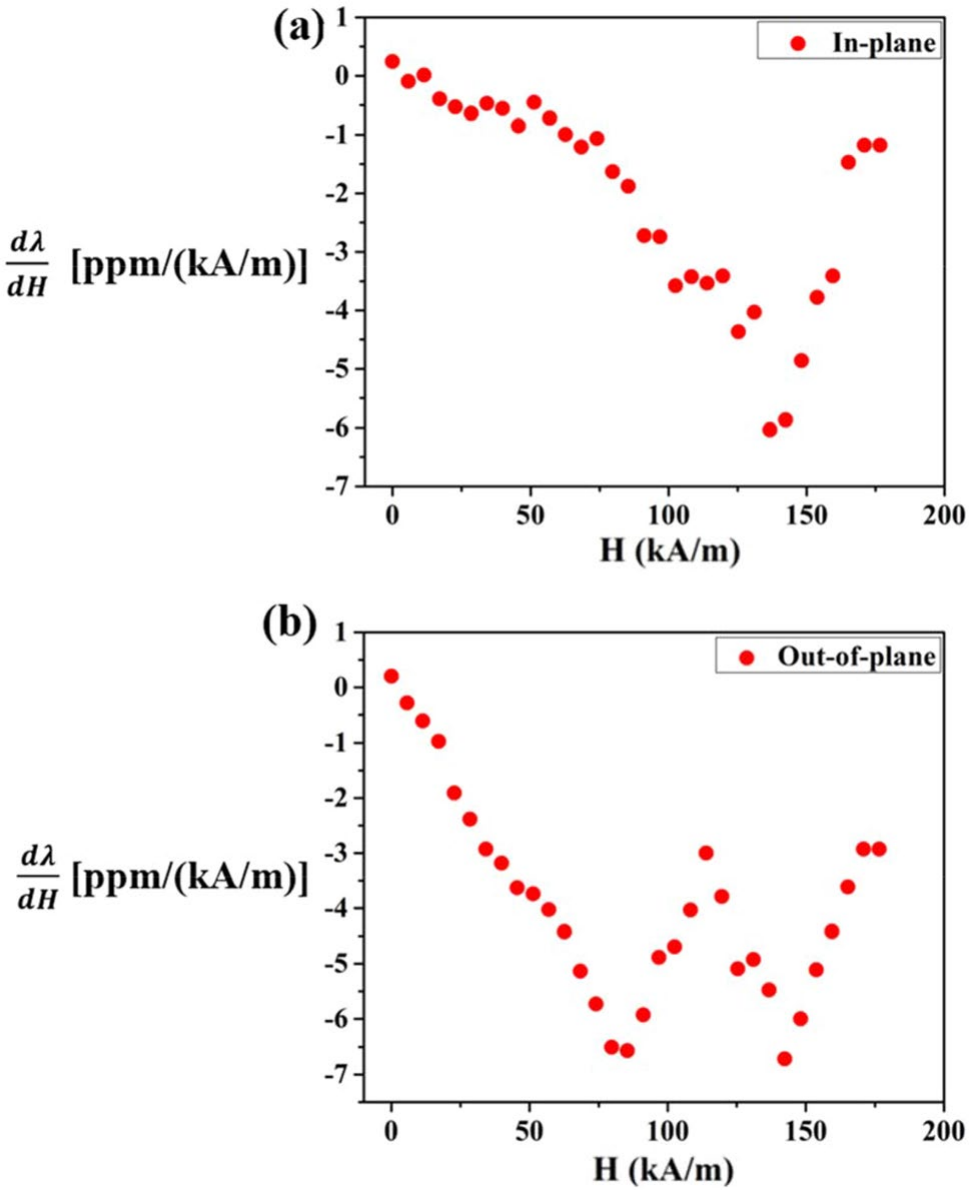
(**a**) The variation of strain sensitivity with field in in-plane configuration. (**b**) Strain sensitivity vs field curve in out-of-plane geometry.

In the spinel crystal structure, the octahedrally coordinated Co^2+^ ions possess an unquenched orbital magnetic moment (~ 0.6 ${\mu }_{B}$) in addition to the spin magnetic moment^22^. Now, in presence of the magnetic field, spin–orbit coupling takes place at the octahedral co-ordination sites and as a consequence of this, high magnetocrystalline anisotropy is originated into the CFO film which leads to the remarkably high magnetostriction in both configurations at room temperature^33^. We have achieved a significant increment, ~ 83%, of maximum magnetostriction value in the out-of-plane configuration with respect to the in-plane one. This considerable enhancement of magnetostriction is ascribed to the increased deflection of the CFO/Si composite when it is subjected to a magnetic field along its thickness (out-of-plane) than along its width (in-plane). In the out-of-plane geometry, the effective surface area of the film exposed to the field is ~ ${10}^{-6}$ m^2^ [${l}_{f} \left(mm\right)\times {w}_{f} (mm)$], whereas, in the in-plane geometry, the exposed surface area is much less, ~ ${10}^{-12}$ m^2^ [${l}_{f} \left(mm\right)\times {t}_{f} (nm)$]. Due to the exposure of the greater surface area to the magnetic field in the former configuration, a comparatively large number of 90° magnetic domains take part in the origin of the magnetostriction of the film. Now, the magnetostrictive stress (${\sigma }_{m}$) developed into the film due to the deformation of domains is much more in the out-of-plane configuration and subsequently, the external torque (${T}_{ext}$) is increased because of its linear dependence on magnetic stress (${T}_{ext} \propto {\sigma }_{m}$). From the working principle of CBM, this increased external torque causes more bending of the CFO/Si composite resulting in enhance of magnetostriction in the out-of-plane geometry. The complete situation is represented schematically in Fig. 4. Besides, it is very much essential to know about the strain sensitivity ($\mathrm{d\lambda }/\mathrm{dH}$) of the film as far as the application is concerned. We have calculated $\mathrm{d\lambda }/\mathrm{dH}$ from our known (λ‒H) data in the 4th quadrant (ascending cycle) and presented the variation of $\mathrm{d\lambda }/\mathrm{dH}$ for the range of magnetic field (0 ‒ ~ 183 kA/m) in both configurations in Fig. 5a,b. Now one can see how the stain sensitivity depends on the magnetic field. In the in-plane configuration, the maximum strain sensitivity, ${(\mathrm{d\lambda }/\mathrm{dH})}_{max}$ = ‒ 6.04 ppm/(kA/m) is found at a field, ${H}_{1}=$ 136.73 kA/m whereas in the out-of-plane configurtion, the maximum strain sensitivity, ${(\mathrm{d\lambda }/\mathrm{dH })}_{max}$ = ‒ 6.72 ppm/(kA/m) is obtained at a field ${H}_{2}=$ 142.43 kA/m. It is not necessary that ${\mathrm{H}}_{2}$ will be larger than ${\mathrm{H}}_{1}$ for strain sensitivity (piezo-magnetic coefficient), although it is expected for magnetization and magnetostriction as a function of magnetic field. As a consequence of these large strain sensitivity values in both geometries, the CFO film has been emerged as a superior candidate in designing various types of room temperature sensors used in aerospace, acoustic industries as well as in MEMS (Micro-Electro-Mechanical Systems) technology.

## Conclusion

In summary, we have prepared polycrystalline CFO film through the pulsed laser deposition system and measured the magnetostrictive strain at room temperature in in-plane and out-of-plane configurations using the optical cantilever beam magnetometer set-up. The magnetostriction varies compressively in both configurations. The film possesses high magnetostrictive strain in both geometries and also shows considerable enhancement of its strain value in the out-of-plane geometry.

## Methods

First of all, CFO nanoparticles were synthesized through the sol–gel technique by adding [Co(NO_3_)_2,_ 6H_2_O] and [Fe(NO_3_)_2_, 9H_2_O] as precursor materials at 1:2 molar ratios in ethylene glycol medium. The sol synthesis procedure has been described in detail elsewhere^10^. The prepared sol was then heated at ~ 190 °C to form drier gel particles. Next, we calcined the gel particles at 450 °C for 5 h to get the desired nanoparticles. After that, the CFO target of 1″ diameter was prepared by pressing the CFO nanoparticles with the help of a hydraulic pellet press. Finally, the target was sintered at 800 °C for 6 h inside a tube furnace to enhance the hardness. Now, this target was used to deposit a CFO film on a double-sided polished n-Si(100) cantilever substrate [length (${l}_{s}$) = 25 mm, width (${w}_{s}$) = 5 mm and thickness (${t}_{s}$) = 130 μm] by pulsed laser deposition system. At first, the pre-cleaned substrate was masked properly at both ends before being mounted inside the PLD chamber. Then, the chamber was evacuated up to a base pressure of 10^–5^ mbar. KrF excimer laser of 248 nm wavelength and 10 Hz repetition rate was employed at energy 400 mJ for the deposition. The substrate temperature was kept at 550 °C, and 0.07 mbar of oxygen pressure was maintained during the deposition. To confirm the formation of spinel phase, we investigated the GIXRD profile of the CFO film in θ–2θ mode over the angular range 30°–70° using PHILIPS X'Pert X-ray diffractometer with Cu ${K}_{\alpha }$ radiation (λ = 1.54 Å). We have carried out the AFM measurement in tapping mode over the scan area (5 μm × 5 μm) to estimate the root mean square (r.m.s) surface roughness of the film. In order to measure the thickness of the CFO film, we captured the cross-sectional view of the CFO/Si heterojunction at a magnification of 50,000 (50 k) through the MERLIN FESEM. We have performed the magnetization measurement of CFO film using the optical cantilever beam magnetometer set-up at room temperature. The magnetization measurement has been performed under the application of ~  ± 40 kA/m magnetizing field along the length (say, along the x-axis) of the cantilever while keeping the sample under a small constant deflecting field along the thickness (say, along the z-axis) of 1.91 kA/m during the experiment. We have used the following relation to calculate the magnetization of the film^26^:

1 $$M= \frac{{Y}_{s}{w}_{s}{t}_{s}^{3}}{2{V}_{f}{H}_{z}}[\frac{1}{3\left(b+a\right)\left({l}_{eff}-b\right)+2b\left(b+a\right)- {a}^{2}}]\Delta$$

where the suffixes "s" and "f" stand for substrate and film, respectively. *Y*, *w*, and *t* represent Young's modulus, width, and thickness, respectively. V and ${H}_{z}$ correspond to the volume and deflecting field. Δ is the deflection of the sample/substrate composite. To measure room temperature in-plane magnetostriction of CFO film using the CBM technique, firstly, we have measured the deflection (Δ) of the cantilever substrate/sample composite by applying the bipolar magnetic field, ~  ± 183 kA/m along the length (${l}_{f}$) and the width (${w}_{f}$) of the film separately. The corresponding deflections and stresses developed in the sample are denoted as {(${\Delta }^{l}$), (${\sigma }_{m}^{l}$)} and {(${\Delta }^{w}$), (${\sigma }_{m}^{w}$)}, respectively. The magnetostrictive stresses along both directions have been calculated with the help of the following equation^26^:

2 $${\sigma }_{m}= \frac{2{Y}_{s}}{9(1+ {\nu }_{s})} \frac{{w}_{s}}{{w}_{f}} \frac{{t}_{s}^{3}}{{t}_{f}} \frac{\Delta }{\left(\beta {t}_{s}+ {t}_{f}\right)[{\left({l}_{eff}-a\right)}^{2}- {({l}_{eff}-b)}^{2}]}$$

Here, ν is the Poisson’s ratio, and β ${t}_{s}$ is the distance between the sample/substrate interface and the neutral plane. The factor β is 1/2 for the unstrained interface of film/substrate heterostructure, whereas 2/3 in the case of strained interface^26,34^. We have calculated the in-plane magnetostriction (${\lambda }_{IP}$) of the CFO film by taking the difference between the in-plane stresses, i,e., (${\sigma }_{m}^{l} - {\sigma }_{m}^{w}$). The related equation describing the in-plane magnetostriction is given by^26^

3 $${\lambda }_{m}= \frac{4}{27} \frac{{Y}_{s}}{(1 + {\nu }_{s})} \frac{(1+ {\nu }_{f})}{{Y}_{f}} \frac{{w}_{s}}{{w}_{f}} \frac{{t}_{s}^{3}}{{t}_{f}} \frac{{\Delta }^{l}- {\Delta }^{w}}{\left(\beta {t}_{s}+ {t}_{f}\right)[{\left({l}_{eff}-a\right)}^{2}- {({l}_{eff}-b)}^{2}]}$$

Here, ${\Delta }^{l}$ (${\Delta }^{w}$) is the deflection of the sample/substrate composite when the field is along the length (width) of the sample. Till now there is no article on the measurement of out-of-plane magnetostrictive strain. For the first time here, we are reporting the magnetostrictive strain of the CFO film in the out-of-plane configuration at room temperature. The finding of magnetostrictive strain in this geometry enhances the potentiality of the film in making different types of sensors operated at room temperature. In order to measure the out-of-plane magnetostriction of CFO film using the CBM technique, firstly, we have magnetized the sample by applying the bipolar field of ~  ± 183 kA/m along with the thickness of the film and recorded the deflection (${\Delta }^{t}$) of the sample/substrate composite. Then the magnetostrictive stress (${\sigma }_{m}^{t}$) developed in the film in the out-of-plane configuration has been calculated using the Eq. (2) and finally, we have taken the difference between ${\sigma }_{m}^{t}$ and ${\sigma }_{m}^{l}$ to determine the magnetostriction in this configuration. The equation for magnetostriction in the out-of-plane configuration would be^26^

4 $${\lambda }_{m}= \frac{4}{27} \frac{{Y}_{s}}{(1 + {\nu }_{s})} \frac{(1+ {\nu }_{f})}{{Y}_{f}} \frac{{w}_{s}}{{w}_{f}} \frac{{t}_{s}^{3}}{{t}_{f}} \frac{{\Delta }^{l}- {\Delta }^{t}}{\left(\beta {t}_{s}+ {t}_{f}\right)[{\left({l}_{eff}-a\right)}^{2}- {({l}_{eff}-b)}^{2}]}$$

**Figure 6:**
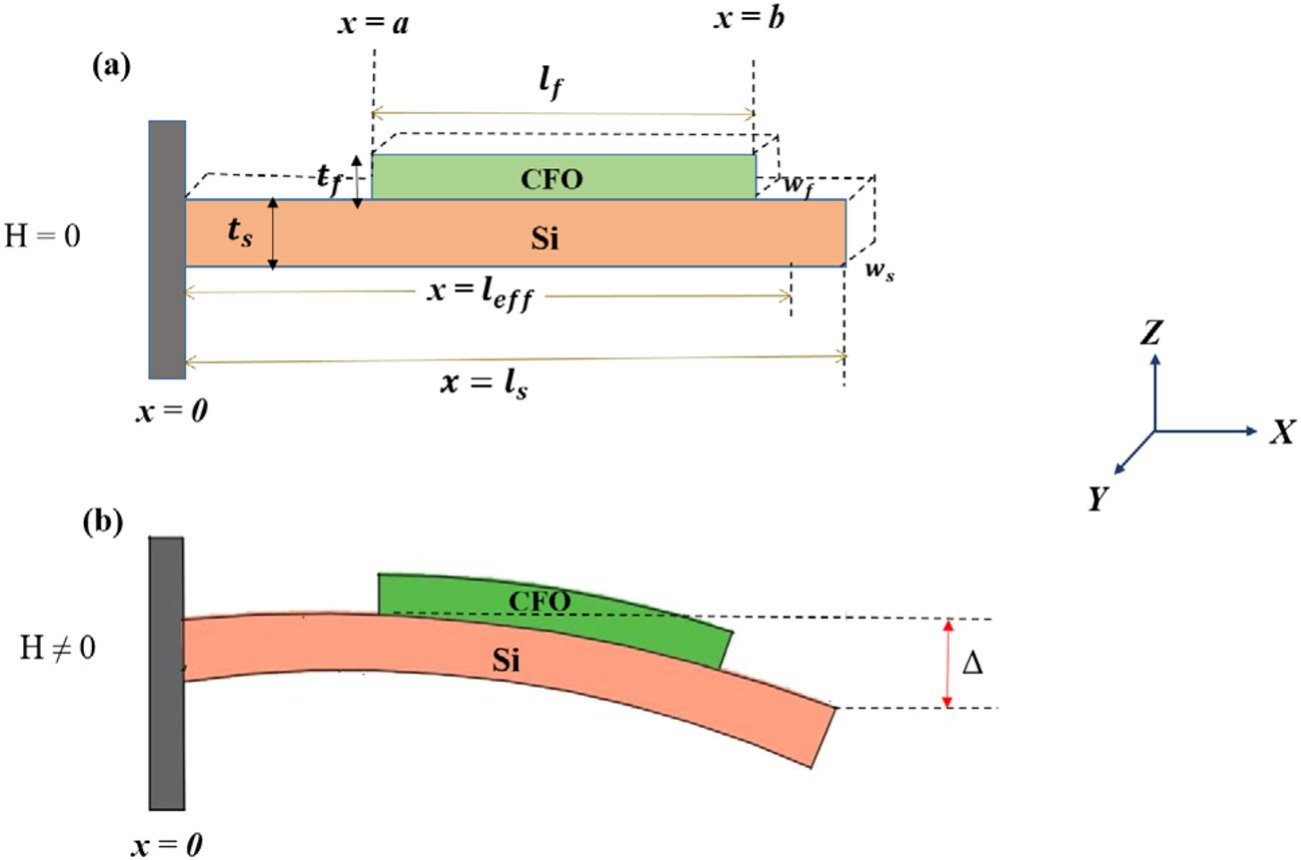
The schematic representation of the geometry of the CFO/Si composite structure with all the essential parameters. (**a**) CFO/Si composite when field is not applied. (**b**) Schematic view of the bending of CFO/Si composite due to the application of the field.

The geometry of the CFO/Si composite structure with all the parameters required for the determination of magnetostriction and magnetization is shown schematically in Fig. 6.
